# Y-Chromosomal Insights into Breeding History and Sire Line Genealogies of Arabian Horses

**DOI:** 10.3390/genes13020229

**Published:** 2022-01-26

**Authors:** Viktoria Remer, Elif Bozlak, Sabine Felkel, Lara Radovic, Doris Rigler, Gertrud Grilz-Seger, Monika Stefaniuk-Szmukier, Monika Bugno-Poniewierska, Samantha Brooks, Donald C. Miller, Douglas F. Antczak, Raheleh Sadeghi, Gus Cothran, Rytis Juras, Anas M. Khanshour, Stefan Rieder, Maria C. Penedo, Gudrun Waiditschka, Liliya Kalinkova, Valery V. Kalashnikov, Alexander M. Zaitsev, Saria Almarzook, Monika Reißmann, Gudrun A. Brockmann, Gottfried Brem, Barbara Wallner

**Affiliations:** 1 Institute of Animal Breeding and Genetics, University of Veterinary Medicine Vienna, 1210 Vienna, Austria; vet.remer@gmail.com (V.R.); elif.bozlak@vetmeduni.ac.at (E.B.); sabine.felkel@boku.ac.at (S.F.); lara.radovic@vetmeduni.ac.at (L.R.); doris.rigler@vetmeduni.ac.at (D.R.); gertrud.grilz@vetmeduni.ac.at (G.G.-S.); GWaiditschka@in-the-focus.com (G.W.); gottfried.brem@bremkg.de (G.B.); 2 Vienna Graduate School of Population Genetics, University of Veterinary Medicine Vienna, 1210 Vienna, Austria; 3 Institute of Computational Biology, Department of Biotechnology, BOKU University of Life Sciences and Natural Resources, 1190 Vienna, Austria; 4 Department of Animal Molecular Biology, National Research Institute of Animal Production, 32-083 Balice, Poland; monika.stefaniuk@iz.edu.pl; 5 Department of Animal Reproduction, Anatomy and Genomics, University of Agriculture in Kraków, 30-059 Kraków, Poland; monika.bugno-poniewierska@urk.edu.pl; 6 Department of Animal Science, UF Genetics Institute, University of Florida, Gainesville, FL 32610, USA; samantha.brooks@ufl.edu; 7 Baker Institute for Animal Health, College of Veterinary Medicine, Cornell University, Ithaca, NY 14853, USA; dm96@cornell.edu (D.C.M.); dfa1@cornell.edu (D.F.A.); raheleh.sadeghi2@gmail.com (R.S.); 8 Equestrian Federation of The Islamic Republic of Iran, Tehran 193954754, Iran; 9 College of Veterinary Medicine and Biomedical Sciences, Texas A&M University, College Station, TX 77843, USA; GCothran@cvm.tamu.edu (G.C.); rjuras@cvm.tamu.edu (R.J.); anas.khanshour@gmail.com (A.M.K.); 10 Center for Pediatric Bone Biology and Translational Research, Texas Scottish Rite Hospital for Children, Dallas, TX 75219, USA; 11 Identitas AG, R&D, 3014 Bern, Switzerland; stefan.rieder@identitas.ch; 12 Veterinary Genetics Laboratory, University of California, Davis, CA 95617, USA; mctorrespenedo@ucdavis.edu; 13 All-Russian Research Institute for Horse Breeding, 391105 Ryazan, Russia; l.v.kalinkova@gmail.com (L.K.); vniik08@mail.ru (V.V.K.); amzaitceff@mail.ru (A.M.Z.); 14 Albrecht Daniel Thaer-Institut, Humboldt-Universität zu Berlin, 10115 Berlin, Germany; saria.marzook.de@gmail.com (S.A.); monika.reissmann@agrar.hu-berlin.de (M.R.); gudrun.brockmann@agrar.hu-berlin.de (G.A.B.)

**Keywords:** horse breeding, foundation sire, Y chromosome, paternal lineage tracing, haplotype, Arabian horse, pedigree, male genealogy, genotyping

## Abstract

The Y chromosome is a valuable genetic marker for studying the origin and influence of paternal lineages in populations. In this study, we conducted Y-chromosomal lineage-tracing in Arabian horses. First, we resolved a Y haplotype phylogeny based on the next generation sequencing data of 157 males from several breeds. Y-chromosomal haplotypes specific for Arabian horses were inferred by genotyping a collection of 145 males representing most Arabian sire lines that are active around the globe. These lines formed three discrete haplogroups, and the same haplogroups were detected in Arabian populations native to the Middle East. The Arabian haplotypes were clearly distinct from the ones detected in Akhal Tekes, Turkoman horses, and the progeny of two Thoroughbred foundation sires. However, a haplotype introduced into the English Thoroughbred by the stallion Byerley Turk (1680), was shared among Arabians, Turkomans, and Akhal Tekes, which opens a discussion about the historic connections between Oriental horse types. Furthermore, we genetically traced Arabian sire line breeding in the Western World over the past 200 years. This confirmed a strong selection for relatively few male lineages and uncovered incongruences to written pedigree records. Overall, we demonstrate how fine-scaled Y-analysis contributes to a better understanding of the historical development of horse breeds.

## 1. Introduction

Since their first domestication approximately 5000 years ago, horses have been inextricably linked to human societies [1,2]. Apart from their economic value and significant impact as working animals, horses have a pronounced cultural heritage value and exert a strong emotional attraction on people. The evolution of horse breeds [3], the relationship among breeds [4], or even the ancestry and influence of single individuals [5] are of particular interest to the scientific and public communities.

Most of our insights into the origins and the genetic compositions of horse breeds rely on studbook records and on genomic studies which are predominantly driven by investigations of autosomal loci or the maternally inherited mitochondrial DNA (mtDNA) (reviewed in [6]). These studies delineated the fact that the domestic horse populations continuously changed in the course of historic development [7] with the most pronounced changes occurring in the past 200 years as a result of intensive selective breeding.

In this recent period, the prevalent strategy to introduce new phenotypes or consolidate traits was and remains the increased use of carefully selected stallions [8,9]. To date, insights into stallion-mediated refinement derived from studbook information indicates that stallions used for breed improvement were often imported from distant regions, that they were extensively shifted between studs, and that their heritage was amplified by their sons and grandsons. This cumulative impact of a stallion led to the establishment of a ‘sire line’. By definition, members of a sire line trace back to a single, often renowned, foundation individual in their paternal line of inheritance. Due to intensive selection of stallions, the predominance of a few sire lines is the rule rather than the exception in intensively managed horse breeds [10,11]. The English Thoroughbreds, with a well-documented closed studbook first published in 1791 [12], provides a compelling example of this sex bias in horse breeding with the tail-male lineages of only three foundation sires being retained today [13,14].

However, the depth and quality of breeding records vary significantly among breeds. Besides the English Thoroughbred studbook, the deepest breed registries, dating back to the 18th century (up to 30 generations back), exist for some Central European breeds (for example [15,16]). The pedigree records illustrate the remarkable influence of Spanish, Arabian, and Thoroughbred stallions on the formation of many modern breeds. Given the eminence of stallions in breeding, a pedigree-independent genetic characterization of patrilines in a breed would be a milestone for the horse community.

The genetic compartment that perfectly matches the paternal lineage in mammals is the male-specific, nonrecombining part of the Y chromosome (MSY). The MSY is inherited as a single linkage group, defined as a haplotype (HT), from a father to his sons. Due to the close association with the patriline, the MSY became the most popular marker in human genetic genealogy [17,18]. In horses, MSY analysis could reveal the influence and origin of breeding stallions, similar to human family history research, or even be useful for forensic applications [19].

Recently, a stable horse MSY HT topology based on slow evolving biallelic markers was defined using next generation sequencing (NGS) technology [20,21,22]. Domestic horse MSY HTs are clearly distinct from those in the Przewalski’s Horse. The most pronounced MSY signature in modern horse breeds is the predominance of a very recently expanded haplogroup (HG; in this context a defined group of closely related HTs). All Central and South European, American, and most East Asian horses investigated to date carried this so-called ‘crown’ HG. The most recent common ancestor (MRCA) of the crown was dated to 1000–2000 years before present [22]. The crown was postulated to be the footprint of Oriental horses [20], but the exact ways and timeframes by which the Oriental HTs were disseminated in the past are not yet fully resolved. ‘Non-crown’ HTs have so far been detected only in Asian horses [21,23,24] and in some northern European breeds [20,25].

The feasibility of MSY patriline tracing with biallelic markers, even among the recently established crown HTs, was demonstrated in the three English Thoroughbred sire lines [22], where deep pedigree information exists back to the 18th century. The HTs carried by the foundation sires of the Thoroughbred were inferred by combining sire line genealogies from pedigrees with MSY HTs ascertained from NGS sequencing data in numerous extant descendants. The three Thoroughbred foundation sires carried an HT in the crown HG ‘Tb’. Moreover, several ‘subline-HTs’, which arose from de novo mutations within the pedigree-supported timeframe, were detected. Those ‘subline-HTs’ could be unambiguously linked to a specific offspring and thus serve as specific markers for the breeding influence of particular stallions on the genealogical scale. The most prominent HT ‘Tb-dW1’, specific to the progeny of the stallion Whalebone (1807), unequivocally delineates the influence of this bloodline on many breeds [20,26,27]. However, apart from the Thoroughbred lines, the MSY HTs for the many other influential stallions and refining breeds are not yet defined.

In this study, we introduce fine-scaled MSY haplotyping in Arabian horses. Selectively bred over centuries by Arab people, the Arabian horse can be considered the oldest horse breed in the world. Arabian horses were used for enhancement in many of today’s breeds, but there are few areas in the world that have not used Arabian bloodlines to refine native horses [8,28]. As shown in a recent study based on ancient DNA [7], a significant influence of Persian horse lines on European and Central Asian stocks is evident from the 7th–9th century onwards. In more recent periods, Arabian horses grew in popularity in the Western world from the 18th century onwards. European nobles and breeders purchased breeding animals in the Middle East directly from Arabian owners or traders and imported them to Europe with the intention to either improve their local herds or breed Arabians outside their region of origin. The popularity of Arabian horses throughout the 19th and into the first decades of the 20th century marks the ‘Arabian wave’ in European horse breeding. In this period, several Arabian horse lineages were established in many countries and today the Polish, Egyptian, French, Russian, British, and US Arabians are among the leading ones. Nowadays, Arabian horses are bred for several types around the world through 63 Arabian pedigree registries [29].

Whereas for centuries Arabian horses were bred in their native based on maternal strains, selective breeding outside the Middle East became focused on sire lines. Consequently, few tail-male lines are retained in Arabian populations today, with the foundation sires originating from different Bedouin tribes, strains, geographical regions, and time periods. Many of the Arabian sire lines are globally active today, and some lines have been re-introduced into their regions of origin [29].

Here we apply fine-scaled Y-chromosomal HT analysis in Arabians for the first time and present what this powerful lineage tracer informs about in terms of the origin, purity, genealogies, dispersal, and influence of Arabian stallions.

## 2. Materials and Methods

A glossary with the definitions for the terms used in the following sections is given in Appendix A Table A1.

### 2.1. Creating a Refined Y-Chromosomal Haplotype Phylogeny

#### 2.1.1. Next Generation Sequencing (NGS) Data

- Whole Genome NGS Data

Whole genome NGS (WGS) data from 118 male horses (117 domestic horses and a single Przewalski’s Horse) mapped to the LipY764 (GCA_002166905.2) Y chromosome assembly were used from a previous study [22]. Among these samples, 114 belong to the crown haplogroup. An Icelandic Horse, a Shetland Pony, and a Przewalski’s Horse were included as outgroups. Details about WGS data are given in Table S1.

- Target Enriched Sequencing Data

**Figure A1:**
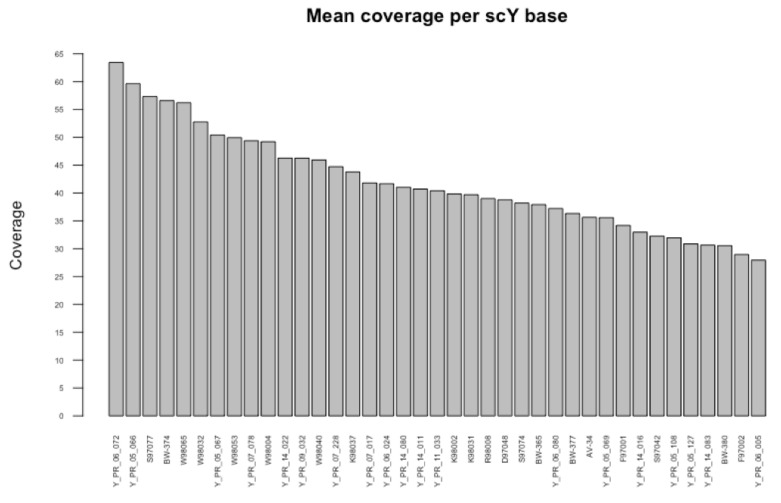
Target-enriched sequencing depth.

We performed Y-chromosome-target enriched sequencing (TES) of 39 males. The horses selected for TES included Arabians, Arabian-influenced breeds, Baroque Type breeds, and Coldbloods (Table S1). Genomic DNA was isolated from the Biosample given in Table S1 for each individual using DNeasy Blood and Tissue Kit from Qiagen, Vienna, Austria. For NGS library preparation and target enrichment, the Custom SureSelectXT low input target enrichment from Agilent, Vienna, Austria, was used. As previously described [22], the LipY764 assembly harbors 5.8 Mb of so-called single-copy Y (scY) regions that were shown to be suitable for unambiguous variant calling. Baits were generated which covered 5.063 Mb of scY regions of the LipY764 assembly, the mtDNA, and some autosomal loci. The 4032 Y-chromosomal bait segments that cover 5.06 Mb (86.7%) of the LipY764 scY regions are shown in Table S2. Indexed libraries were generated, and the enrichment was performed according to the protocol supplied by Agilent. Libraries were pooled and sequenced on two Illumina NextSeq550Medium PE150 runs (San Diego, CA, USA) (Table S1) at Vienna Biocenter Core Facilities, Vienna. Only reads mapping to the Lip764 assembly (see below) were analyzed in this study (Figure A1).

#### 2.1.2. Data Analysis

- Variant Ascertainment

TES data were demultiplexed, adapters were removed with AdapterRemoval [30], and reads were trimmed with ReadTools [31]. Versions for programs used are provided in Appendix B Table A2. Adapter-free and trimmed reads were mapped to the LipY764 assembly (GCA_002166905.2) using bwa [32] with the parameters bwa aln −n 0.02 −l 200. Unmapped reads, PCR duplicates, and low-quality mappings (−q 20) were filtered out with samtools [33]. Variant calling was performed on the 39 TES mappings using GenomeAnalysisTK’s [34]. First, gvcf files were produced by HaplotypeCaller, and then these files were merged with GenotypeGVCFs. Only the variants in the scY windows (these regions in LipY764 were defined in [22]) were considered for further analysis using bedtools [35]. We filtered out reference errors, sites called heterozygous only and variants with multiple alternatives. Variants with a read depth <3 and genotype quality <5 in more than 10% of the samples were excluded from further analyses using a custom python script (available from the authors on request). Furthermore, we just retained variants that were called in 75 % of the samples. We then merged the final variant list with the Y-chromosomal variant panel from [22]. All variants that were first ascertained in the TES data were visually checked in the IGV browser [36].

- MSY Haplotype Tree

**Figure A2:**
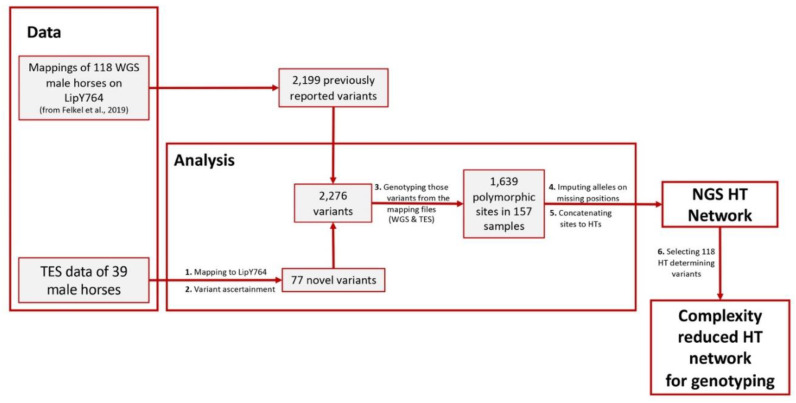
NGS data analysis pipeline. Two NGS data sets were merged to produce the final structure: mappings of 118 WGS males to LipY764 from a previous study and newly generated TES data of 39 males. The 77 novel variants ascertained in the TES dataset were merged with 2199 previously defined variants resulting in a total of 2267 variants. Those variants were genotyped in the mapping files, missing positions imputed, and HTs constructed by concatenating the polymorphic sites. HTs were visualized in a network format. A total of 118 variants was selected for genotyping. Detailed descriptions of the workflow, including programs, and parameters, is provided in the Material and Methods section.

The allelic state of 2276 MSY variants (2199 variants published [22] and 77 variants newly ascertained from the target enriched data) were genotyped in the 118 WGS sequenced and the 39 target-enriched samples sequenced using freebayes [37]. In total, 1639 MSY variants were polymorphic in our genotyping panel. In order to impute sites with missing calls in NGS data, which occurred either because of low coverage in WGS data or because the site is not covered in TES data, the samples were first clustered into haplogroups based on previously described haplogroup-determining variants [22]. Missing calls were manually added by introducing the allelic state observed in samples of the same group (strategy described in [38]). After gap filling, the states of the 1639 variants were concatenated to construct MSY HTs. Variant ascertainment and haplotype reconstruction strategy is outlined in Appendix B Figure A2. The Przewalski’s Horse was used to infer the ancestral state for the variants that are polymorphic in the domestic horse. The topology and frequencies of the MSY HTs were visualized with the program Network [39]. For HT nomenclature, the first four letters were kept consistent with our previous studies [20,22], followed by a successive letter/number according to human guidelines for MSY HTs [40]. The first four-letter code is deliberately not in alphabetical order but rather informs in which breeds/populations the HG/HT was first described. For details see Appendix C Table A3.

### 2.2. MSY Haplotyping

#### 2.2.1. Creating the Backbone Structure

**Figure 1:**
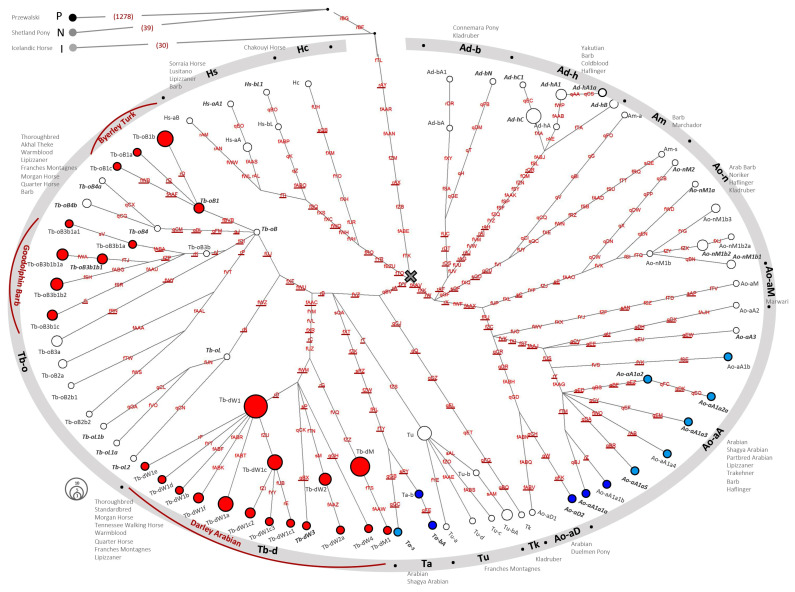
NGS HT Network. The MSY HT network from 118 WGS sequenced males and 39 TES males, based on a total of 1639 variants (281 crown, 1358 outside the crown). HTs are indicated as circles, with circle size being proportional to frequency. HT-IDs and sample information are provided in Table S1. HTs first described in this study based on TES data are shown in bold. Variants are indicated on branches and underlined when selected for genotyping. The position of the crown MRCA is marked with a cross. The 14 crown HGs are indicated in the outer circle, with the breeds listed beside them. Blue HTs were detected in Arabians, and light blue HTs were detected in a horse that traced back to an imported Arabian in the paternal lineage. The signatures of the three founders of the English Thoroughbred (Tb-oB1, Tb-oB31, and Tb-d) are marked with red lines.

Out of the 290 crown variants, we defined 118 markers (110 Single Nucleotide Variants (SNVs), seven short insertions/deletions (Indels), and one short tandem repeat (STR)). The selected markers were checked visually in the IGV browser [36], by comparing the site in multiple samples in parallel. The markers are underlined in Figure 1 and listed in Table S3. These were catenated to generate a simplified crown HT tree (Figure S1, HTs in Table S4). This tree served as a backbone for MSY HT screening.

#### 2.2.2. Haplotype Determination

For MSY haplotyping, genomic DNA from male horses was isolated using nexttec, Hilgertshausen, Germany. DNA Isolation Kits, using the biological material given in Table S5. To genotype 117 variants (SNVs and Indels), PCR-based KASP™ (Kompetitive Allele-Specific PCR) genotyping technology (KASP™, lgcgroup.com) was used. KASP assays were designed and KASP™ screening was performed on a CFX96 Touch™ Real-Time PCR Detection System using the standard protocol provided by the supplier (LGC, Berlin Germany). Cluster plots were analyzed using Bio-Rad CFX Manager 3.1 (Biorad, Vienna, Austria). Samples with known allelic states (DNA from sequenced individuals, and DNA from a Przewalski’s Horse, a Shetland Pony, and an Icelandic Horse) were included as positive controls in each run. DNA from females and water controls were used as negative controls.

To genotype the amplicon length of the STR fBVB on an ABI 3130xl Genetic Analyzer, one primer was labeled with FAM fluorescent dye (fwd_FAM: ACAACCTAAGTGTCTGTGAATGA; rev: CCCAATAATATTCCACTGCGTGT). PCR was performed on a 20 µL volume containing 0.4 µM of each primer. The DNA was initially denatured at 95 °C for 5 min, followed by 35 cycles of 30 s at 95 °C, 40 s at 58 °C annealing temperature, 40 s at 72 °C, and a final extension of 30 min at 72 °C. Alleles were sized relative to the internal size standard using GeneMarker® (Softgenetics, State College, USA).

Genotyping was performed in a consecutive manner. First, we confirmed that each sample belonged to the crown haplogroup by testing for the key variants rAY and rAX. Second, we clustered them into one of the major crown clades T, A, or H by testing for rA, rW, and fYR (see Figure 1). We then genotyped each sample for variants that determine the haplotype in the respective clade and imputed the ancestral allele in variants informative in the other clades. For the 38 individuals included in the MSY haplotyping approach that had NGS data available (Table S5), the allelic states of the 118 defined variants were extracted from the freebayes genotyping results (Table S6).

Haplotype networks were generated using the program Network [39]. The HT network was redrawn with the program draw.io. Not yet fully NGS-ascertained (most possibly internally branching off) HTs are marked with an asterisk and are outlined in the network with a dashed line branching off at the respective internal node (see Figure S1).

#### 2.2.3. Datasets

- Globally Active Arabian Sire Lines

A dataset was assembled with sire lines that are presently active in the occidental Arabian horse population. Tail-male line evaluation from pedigrees of present-day breeding stallions formed the basis for sample collection. We first reconstructed the paternal genealogies of hundreds of horses using pedigree information provided by breeding associations, studbooks, or by reconciling results from multiple databases (listed in Table S5) and stored the tail-male line in a string format. Based on these analyses, we chose two to three present male descendants of each sire active in the 1970s and collected a sample (biological material given in Table S5) for genotyping. We further included sire lines that evidently originated from imported Arabians but survived only in other breeds, like the Shagya Arabian, the Trakehner, and the Lipizzaner. With this strategy, we attempted to obtain the progeny of as many imported foundation sires identified and also represent their sublines, while minimizing oversampling of certain lines. Furthermore, we selected the samples from different breeding registries if a line was universally represented. However, for some lines, only a few samples or sometimes even a single sample were available. In total, our final sample set of 145 males comprised 81 registered Arabian Horses, 31 Shagya Arabians, 5 Partbred Arabians, 4 Fredriksborg Horses, 1 Kladruber, 2 Knabstrupper, 1 Lewitzer, 11 Lipizzaner, 1 Pintabian, 5 Trakehner, and 3 Warmbloods. Tail-male line reconstruction as a string format is outlined in Table S6, where the most recent ancestor given for the sampled horse was born no later than 1995, in order to protect the anonymity of the sample analyzed.

We need to state here that seven Arabian horses and four Akhal Teke were observed to carry the Tb-dW1 haplotype. This HT was previously attributed to a recent admixture with Thoroughbreds [20,22,27]. These horses were omitted from further analysis as the goal of this work was to focus solely on the Arabian Y-chromosomal lineages.

- Sampling Local Arabian Lines and Other Populations

A dataset of 35 Arabian horses, 8 from Iran and 27 from Syria, representing local lines was collected. The samples derive from three independent sampling projects, and pedigree information was available only for a few animals. If the pedigree information of a sample existed, it confirmed that the horse did not trace back to any globally active line. Furthermore, twelve Turkoman horses from Iran and 28 Akhal Teke horses from several countries were used. Finally, 24 Thoroughbreds and 15 Trotters, representing the three foundation sires of the Thoroughbred were included in the dataset. A full description of this dataset including sampling information is given in Table S5.

## 3. Results and Discussion

### 3.1. A Refinement of the Crown HT Structure from NGS Data

In order to perform MSY tracing of sire lines in modern breeds, one needs to distinguish among the very recently established crown HTs with informative markers. Therefore, we first generated a refined topology of the HTs within the crown HG based on NGS sequencing data. In total, 115 HTs from whole-genome sequenced males which were confirmed as crown haplogroup carriers in a previous study [22], were analyzed together with TES data from 39 males generated in the course of this study. The TES data covered the 5 Mb scY region of the MSY with a mean coverage of 41.95 × (Table S1 and Figure A1). While the whole genome sequenced sample collection from the previous study consisted mainly of Sport horses and Thoroughbred-influenced breeds, the horses selected for TES represented Arabians and Arabian-influenced breeds as well as some Baroque Type breeds (such as Lipizzaner, Kladruber, Friesian), Coldblood, Iberian breeds, and some Barb horses. A Shetland Pony, an Icelandic Horse, and a Przewalski’s Horse, previously determined as ‘non-crown’ HT carriers [22] were included as outgroups. The 157 males with NGS data analyzed in this study, including breed, sire line, and raw data information are listed in Table S1. The LipY764 shotgun assembly, which allows unambiguous MSY variant calling on a total length of 5.8 Mb scY regions, was used as reference. We first ascertained variants in the 39 TES samples and retained 177 variants after the filtering steps described in the Material and Methods section. Out of those, 18 did not pass the IGV quality check. From the 159 remaining variants, 82 (53.4 %) were previously described in the WGS dataset and 77 variants (67 SNVs and 10 Indels) were newly ascertained in this study (q-variants). The results from variant ascertainment in TES data in .vcf format are shown in Table S6. 

We then combined the newly ascertained variants with the 2192 MSY specific variants ascertained in [22] and genotyped those in the full dataset (WGS and TES data; see Material and Methods and Figure A2). We detected 1639 variable sites in the 157 males, and those variants are listed in Table S6. The majority of variants (1278 (78%)) separated the Przewalski’s Horse HT from the domestic horses. The two ‘non-crown’ domestic horse HTs in our analysis panel, N (the Shetland Pony) and I (the Icelandic Horse), had 39 and 30 private variants, respectively, and were separated from the crown lineages by nine shared variants. A total of 281 variants (259 SNVs, 20 Indels, 2 STRs) was detected among crown haplogroup carriers. As mentioned above, 77 variants (26.6%) were newly ascertained in this study (q-variants), whereas the remaining 212 crown variants (73.4%) were previously described (r-, s- and f-variants in [20,21,22,26]). The 281 crown variants distinguished 86 HTs. Reconstructed HTs are shown in Table S6. The sequential order of mutations on the nonrecombining MSY allowed a straightforward reconstruction of the HT phylogeny under the principle of parsimony. The resulting topology drawn as a network with variants given on branches is shown in Figure 1. The strictly hierarchic evolution of the MSY HTs is also reflected in the HT nomenclature (for details see Material and Methods, Table A1).

The refined phylogeny confirmed the polytomy of the crown HTs proposed previously [22] and substantiated the three major crown clades H, A, and T (Figure 1). The extended dataset further revealed that the three major clades clearly split into subclades. Hence, the crown HT phylogeny forms a pronounced star-like structure. This observation substantiates the previous hypothesis that the crown HTs are the signature of a remarkable expansion of a population harboring the basal HTs of the crown’s sublines. Within the crown, we define 14 HGs. In Figure 1, we mark the MSY footprint of the three Thoroughbred lineages, which were defined previously [22] as HG Tb-d for Darley Arabian, subhaplogroup (sHG) Tb-oB3b1 for Godolphin Barb, and sHG Tb-oB1 for Byerley Turk.

The Coldblood horses in our dataset grouped primarily into HG Ad-h, while Iberian, Baroque Type, and North African horses, as well as British Ponies, were distributed across several HGs. The five Arabian horses (one each from the Saklawi, Bairactar, and Krzyzyk sire lines, two without pedigree information) clustered together with the seven horses from other breeds (Shagya Arabian, Partbred Arabian, Lipizzaner, Trakehner) that trace back to an Arabian foundation patriline (Siglavy senior (three), Siglavy Bagdady, Ibrahim, Ilderim, and Shagya) into HGs Ao-aA, Ao-aD, and Ta. Each of the twelve horses with Arabian ancestry carried a unique HT. Most Arabian HTs clustered into Ao-aA (eight), followed by Ta (three), and Ao-aD (one). The clustering is shown in Figure 1 and details about samples including breed, sire line information, and information on sequencing data are provided in Table S1. The refined crown topology is a prerequisite for informative sire line investigation in Arabians and other modern breeds. In Felkel et al. 2019 [22], the MRCA of the crown was dated to approximately 1500 years before present. Hence, to accumulate variation, only approximately 190 sequential generations are between the MRCA and present crown carriers when assuming a mean generation interval of eight years [41]. In view of this short evolutionary period, the revealed resolution of crown HTs based on biallelic markers is a remarkable achievement.

### 3.2. Allocation of the MSY Signature in Arabian Sire Lines

In previous studies, Arabian horses were roughly grouped into the crown HGs Ao, Ta, and some racing Arabians into Tb [20,22,27]. The HTs now detected in sequenced males of Arabian origin indicate three HGs (Ao-aA, Ao-aD, and Ta) as being characteristic of Arabian ancestry. However, this finding builds on only twelve sequenced individuals which represent only a few sire lines. Hence, we performed further investigation by genotyping HT-determining variants in a more comprehensive Arabian dataset. For this approach, we selected 118 variants (116 in the crown and the two crown-determining variants, rAY and rAX) to build a complexity-reduced ‘crown-backbone’. In the variant selection, we ensured that the proposed Arabian lineages as well as the Thoroughbred HTs were fully traceable. For HGs that were detected only in breeds other than Arabians and Thoroughbreds (for example Ad-h, Ad-b, Am, Ao-n, Hs, and Hc), we chose fewer variants that would allow the inference of the major clusters but not a fine-scaled HT analysis yet. Selected variants are underlined in Figure 1 and listed in Table S3; the resulting backbone structure is given in Figure S1.

In order to delineate the MSY HT signatures of Arabian horses, we started with Arabian sire lines that became established in Europe based on imported Original Arabians. Many of these lines remain active on a global scale. For horses of these lines, high-quality pedigree information exists, which allowed a systematic representative sampling. Hence, a reconstruction of the tail-male line from the pedigree of registered horses was integrated into the sample selection process (see Material and Methods). Finally, the Arabian HT screening panel consisted of 145 males; 81 registered Arabians from leading breeding populations around the globe, and 64 registered horses from European breeds which trace back to an Arabian stallion imported into Europe in their paternal lineage. In total, the dataset (termed as ‘occidental Arabian lines’) comprised the legacy of 26 Arabian sires that were either imported to Europe from the Middle East during the 19th and 20th centuries or Egyptian foundation stallions. These 26 stallions originated from various Bedouin tribes, represented several strains, and covered more than a 100-year timespan. The first stallion transferred to Europe was Siglavy db, imported in 1814 to Austria, and the latest was Kuhailan Afas, imported in 1931 to Poland (Table 1, Full dataset Table S5).

**Figure 2:**
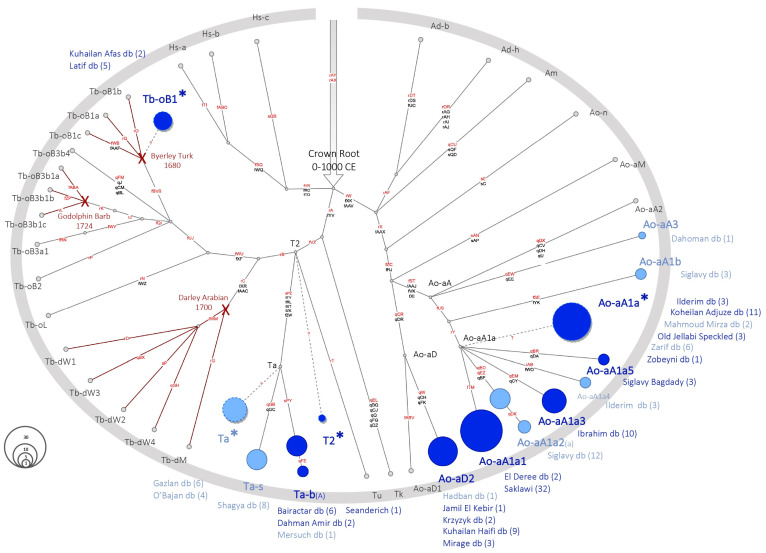
MSY HTs in Arabian sire lines. Simplified crown HT network based on 118 variants. Genotyping results from 145 males are shown in blue circles with size proportional to frequency. Details on samples are given in Table 1, and Table S5 (samples are indicated in Column J; Foundation sires shown in Column AI). Foundation sires are shown for each HT, with the number of samples for each line in parenthesis. HTs/foundation sires that are only active in breeds other than Arabians are given in light blue. Genealogies of the English Thoroughbred founders are outlined with red crosses and the thoroughbred specific subhaplogroups with the branches in red.

We performed MSY haplotyping as described in the Material and Methods sections and first confirmed that all selected MSY variants were informative (details on typing performance are provided in Table S3). Among the 145 males descending from 26 foundation sires, we detected 16 HTs, and all belonged to the crown haplogroup. Results are shown in Figure 2 and Table 1 (full information about the samples is provided in Table S5). We need to point to a limitation of the genotyping approach. Due to ascertainment bias, we might miss the markers determining potentially private HTs in not yet sequenced lines. As the HTs of those lines are not yet fully resolved, they were placed onto internal branching points of the backbone topology. We indicated those HTs with an asterisk (*) in their HT identifier. For example, the horses carrying HT Ta* clustered definitively into the branch Ta (they carried the derived allele for markers sPZ, fTY, fRL, fXT, fZK, and fZW), but they did not group with Ta-s or Ta-b (they carried the ancestral allele for marker qGB, qGC, and sPY). Sequencing Ta* carriers is needed in order to resolve their topology. A description of the HTs is provided in Table S4 and in the Appendix C Table A3.

Overall, most samples (137 out of 145) were grouped into one of the three HGs anticipated as Arabian by the NGS sequenced samples—Ao-aA, Ta, and sHG Ao-aD2. All three HGs were detected in sire lines active in today’s registered Arabians, as well as in lines that survived only in Shagya Arabians or other Arabian influenced breeds (like the Lipizzaner, Kladruber, or Trakehner). Ao-aA was the most common HG seen in 92 samples from 12 sire lines. This HG also had the highest number of accurately defined HTs (9). Among the Ao-aA carriers, most (88) clustered into sHG Ao-aA1a, which determines this sHG as the most pronounced Arabian signature. Six sire lines were placed on the basal *HT Ao-aA1a*, which suggests that we still underestimate HT diversity in this clade.

In addition to the affirmation of the three HGs suggested by the NGS-sequenced horses, genotyping revealed alternative clustering in three lines. A single horse from a Spanish line (foundation sire Seanderich) was grouped to the internal branching point T2*, which indicates a private, not yet resolved, HT in this line. It is worth noting that, in two sire lines (after Kuhailan Afas via Probat, 1975 and Latif via Denouste, 1921; see Table S5), we detected Tb-oB1 (Figure 1 and Table 1). The Tb HG was previously attributed to Turkoman horses [20] and genealogical reconstruction revealed that Tb-oB1 was carried by Byerley Turk in 1680 [22]. In the lines after Byerley Turk, we could only ascertain subline-HTs for lines active in warmbloods (Tb-ob1a-c; [22]). The Byerley Turk Thoroughbred after Djebel, 1937, implemented in the TES set (Y_PR_11_033, Table S1) carried the unaltered foundation HT Tb-oB1. Hence, for the Byerley Turk line, we could not define a long-standing informative lineage-specific marker like, for example, Tb-dW1, which is a subline HT and a unique signature of the Thoroughbred lineage after Whalebone (1807). Considering the early emergence of Tb-oB1 (it was already carried by Byerley Turk in 1680), we expect to underestimate the HT diversity in this sHG in other breeds, due to ascertainment bias. Hence, Tb-oB1* should be the most appropriate term for this sHG at the moment. From the current state of knowledge, we cannot infer explicitly whether ‘Kuhailan Afas and Latif Arabians’ carry Tb-oB1* because of an undocumented Thoroughbred influence in their tail-male line or through another scenario. Both sire lines are well-known for success in flat racing, and the interbreeding of Thoroughbreds in racing Arabians has been shown recently [27]. In particular, a successful ‘Arabian’ racing stallion imported from Syria to Egypt was fathered by the Thoroughbred Temeraire, 1905 from the Byerley Turk line born in Ireland [42]. However, Tb-oB1* in Kuhailan Afas and/or Latif lines could also be an autochthonous Arabian HT. Among the strain types bred by the Bedouins (Kuhaylan, Saglawi, Abayyan, etc.), the Muniqi strain was the racing type [42,43,44]. Muniqi Arabians were phenotypically more similar to Thoroughbreds, and Darley Arabian was described as a Muniqi type Arabian [42]. Hence, Tb-oB1* could have been segregated in Muniqi Arabians without Thoroughbred input and was introduced with horses imported for flat-racing [45].

### 3.3. Horse MSY Haplotyping in Arabian Horses on the Genealogical Scale

In humans, Y-chromosomal haplotyping is a valued system for combining genetics with ancestry information [17]. Personalized characterization of sire lines can allow the tracing of the breeding influence of particular patrilines, or to check for correctness of pedigree records over several past generations. In Felkel et al. 2019 [22], we genetically redrew the three Thoroughbred sire lines. The power of MSY-analysis to detect wrong assignments in the pedigree that occurred multiple generations back in time was exemplified by addressing a historic dispute concerning the wrong paternity assignment of the Thoroughbred stallion Galopin born in 1872.

In the occidental Arabian collection, paternal line information was available for all 145 individuals (information as a string in Table S5). For 20 foundation sires, we had more than one descendant analyzed. In most lines, MSY clustering followed the expectation, and the members of a particular sire line clustered into the same HT/*HT. Some HTs (Ao-aA1a2, Ao-aA1b, Ao-aA1a3, Ao-aA1a4, Ao-aA1a5, Ao-aA3, and Ta-s) were detected only in a single line, whereas others, in particular the not fully resolved *HTs, harbored several lines. Worth mentioning is HT Ao-aA1a1, which was almost private for the seminal Egyptian Saklawi line, after Nazeer, 1934. Apart from the Saklawi line, Ao-aA1a1 HT was only found in two males after El Deree, another Egyptian foundation horse.

We further revealed that four variants (qDK, qFE, rAB, and fWO) occurred recently within the timeframe of pedigree documentation. These mutations define three de novo subline-HTs (Ta-bA, Ao-aA1a2a, and Ao-aA1a4), which are unique for sublines of certain sire lines (details about HTs and lines are provided in Table 1).

In some genealogies, the MSY pattern did not fully agree with the recorded paternal lineage. We observed two very distinct HTs in the sire line after Siglavy db (Ao-aA1a2 and Ao-aA1b) and also in the lines after Ilderim db (Ao-aA1a and Ta-b). Those findings cannot be explained by de novo mutations but rather are a clear sign that more than one stallion contributed to those lines.

**Figure 3:**
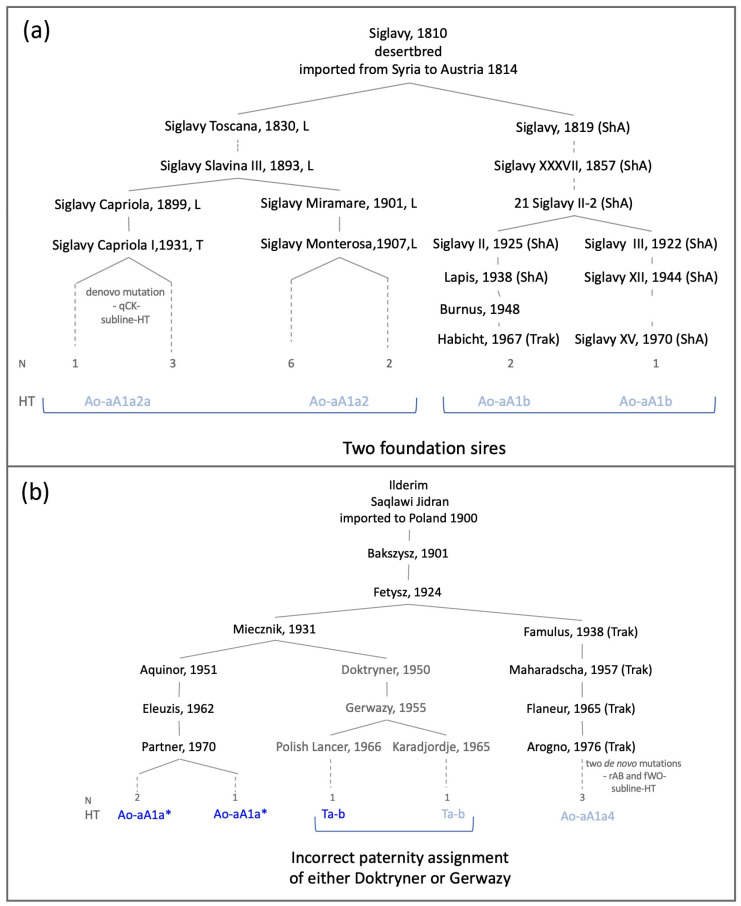
Genealogical cases. (**a**) Paternal genealogies of 15 genotyped male horses after Siglavy, 1810. (**b**) Paternal genealogies of eight genotyped male horses after Ilderim db. Dotted lines indicate that at least one generation is omitted. Abbreviation of horse breeds other than Arabian is given by: L = Lipizzaner, ShA = Shagya Arabian, Trak = Trakehner, AA = Anglo Arabian. The number of genotyped horses and HTs is listed on the bottom (dark HTs were detected in Arabians, light blue HTs in other breeds). The complete tail-male line reconstruction is provided in Table S5.

As shown in Figure 3, we detected two distinct Arabian HTs in horses tracing back to the imported Arabian stallion Siglavy Senior (‘Schwarzenberg’), 1810. The Lipizzaner and Kladruber lines after Siglavy Slavina III, 1893 carried Ao-aA1a2 and the subline-HT Ao-aA1a2a, whereas Shagya Arabians and Trakehner after 21 Siglavy II-2, 1909 grouped distantly into HT Ao-aA1b (Figure 3). Both HTs were not detected in any lines other than the Siglavys (Figure 1). Hence, our findings indicate that the Siglavy sire lines in Lipizzaner and Shagya Arabians were founded by paternally unrelated horses. Confusion may have arisen as a result of homonymous names. A second Siglavy line, founded by the Arabian stallion Siglavy IV db, 1819 (imported 1825), was active in parallel to the Siglavy line after Siglavy Senior (‘Schwarzenberg’), 1810 in the studs of the Habsburg Monarchy [46].

The inconsistency in the lines after Ilderim db suggested a different scenario. As shown in Figure 3, the HT detected in the progeny of the Polish Arabian stallion Doktryner, 1950 via his son Gerwazy, 1955 was Ta-b. This did not concur with the HTs detected in other descendants of Doktryner’s grandfather Fetysz, 1924 via Aquinor, 1951 and Maharadscha,1957 in Arabians (Ao-aA1a) and the Trakehner (subline-HT Ao-aA1a4). A recent inaccurate paternity assignment of either Doktryner or Gerwazy is the most probable explanation.

HT Ta-b has so far been detected only in two Arabian sire lines bred in Poland, Bairactar and Dahman Amir (Table 1). Among the Arabian stallions active in 1949 were the Klemensow Stud, where Doktryner was bred, and the three Arabian stallions used in 1954 at Michalow stud, where Gerwazy was bred; the only member from a Ta-b sire line was Amurath Sahib (1932) after Bairactar db [47]. MSY HTs provide an indication that Amurath Sahib, active at Klemensow Stud, was the biological father of Doktryner.

As exemplified here, MSY lineage tracing revealed genealogical insights about Arabian sire lines. However, the explanatory power depends on an accurate assignment of line-specific HTs. The HT structure of several Arabian lines is not yet sufficiently resolved, which impedes the concise delineation of their paternal genealogies. Ascertainment of more informative markers by sequencing the MSY in an extended set of samples will push MSY analysis towards a forensic tool for redrawing paternal lineages of horses in the future. Moreover, implementing markers with divergent mutation rates [48], for example, adding MSY STR markers, should allow a better discrimination of recently emerged lineages and answer questions about relatedness levels that differ in time depth.

### 3.4. On the History of Arabian Horse Breeding beyond Pedigrees

The combination of MSY topology and pedigrees could enlighten the ancestry and relationship among foundation sires beyond pedigree documentation. From the occidental lineages, we considered Ao-aA, Ao-aD2, and Ta as typical Arabian and Tb-oB1* and T2* as tentative Arabian. However, those populations went through a severe male bottleneck, as our panel originates from only 26 imported stallions. According to several studies, registered Arabian horses in the Middle East show expanded genetic and phenotypic diversity in comparison to the global Arabian bloodlines [27,49,50,51]. The existence of genetically diverse populations of Arabian horses in the Middle East today could also be reflected in more diverse MSY patterns.

**Figure 4:**
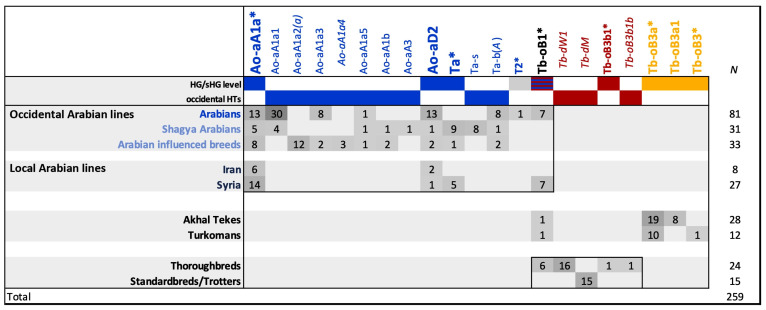
MSY Haplotypes in globally active Arabian lines, Middle Eastern Arabians, and other breeds. Haplogroup (bold) and haplotype distribution in breed or breed groups in absolute numbers *(N* = total number). Thoroughbred HG/HTs are marked in red, Arabian in blue, and Akhal Teke/Turkoman in yellow. The Tb-oB1* subhaplogroup was detected in Thoroughbreds, Akhal Teke/Turkoman, and in a small subset of Arabians, but the ancestry of horses carrying this haplogroup remains unresolved.

Therefore, we extended our Arabian sampling towards local Middle Eastern Arabian populations and genotyped 35 Arabian males, 8 from Iran, and 27 from Syria, with no recently reintroduced occidental lines included. As shown in Figure 4 (full data in Table S5), the HGs detected in Middle Eastern Arabians overlap with the HGs observed in the occidental lines.

Autochthonous Middle Eastern populations clearly substantiated the Arabian MSY signature (Ao-aA, Ao-aD2, Ta, and, presumably, Tb-oB1*). Mainly basal *HTs were observed in the Middle Eastern samples, and we expect them to carry new HTs not yet ascertained via NGS. We did not detect any of the recently established HTs in Middle Eastern populations, a finding that was expected, as those HTs were ascertained in occidental lines through NGS and, accordingly, are specific markers for those lines. The similar MSY HGs in occidental and Middle Eastern Arabians support the hypothesis of a discrete shared origin with recent divergence, a scenario that was proposed from microsatellite analysis [51,52]. 

The grouping of the 28 Akhal Teke and 12 Turkoman males highlights their clear distinction from the Arabians. Most of the samples from those breeds were grouped distantly from the Arabians and into sHG Tb-oB3. This sHG seems, so far, to be private for Akhal Teke and Turkomans, and it shares a more recent ancestry with the Thoroughbred than all Arabian HGs. However, as shown in Figure 4, sHG Tb-oB1*, which was found in two occidental lines, was detected in all comparison datasets. The prominent occurrence of Tb-oB1*, ranging from globally active lines and Middle Eastern Arabian lines from Syria to Akhal Tekes, Turkomans, and Thoroughbreds fuels reflection about scenarios that could have led to this widespread distribution. Together with the other Arabian HTs (Ao-aA, Ao-aD, and Ta), Tb-oB1* could have been typical of autochthonous Arabian horses from the Nejd highlands. Selection and genetic drift could have led to haplotype frequency differences among different strains and areas. In this scenario, Tb-oB1* may have been distributed from the Arabian plateau during the migrations of the Bedouins. Alternatively, Tb-oB1* could also be of Turkoman origin. The influence of Turkoman horses on Arabian horses, albeit rather undesirable from the standpoint of Arabian horse breeders, may have occurred during the Ottoman Empire (from the 15th to the beginning of the 20th century) and the Wahhabi Wars (during the beginning of the 19th century), through the use of units mounted mainly with stallions [53]. Such admixture may also have happened earlier, at the time of the Crusades, when Turkish soldiers came to Syria together with their horses [54]. In this context, Nissen [55] pointed to a combination of Jilfan mares and Turkoman stallions, from which the Muniqi type might have developed. Admixture with Turkoman horses was also postulated for Muniqi-type Arabians by Carl Raswan [42]. Considering the wide distribution of Tb-oB1*, the least likely, albeit possible, scenario is Byerley Turk bloodlines were recently introduced into the Arabian and other horse bloodlines in the Middle East. With the current state of knowledge, we cannot confirm or reject one or a combination of several scenarios. 

Further investigation, based on extended sampling and more sensitive Y-chromosomal markers, is needed to fully resolve the direction of dispersal and influences that happened in the past. We must also keep in mind that the HTs seen in Arabians today are remnants that survived selection through human breeding decisions. Implementation of genetic information from historic remains is now possible [56], and such data will contribute to fully disentangling the origin of present Arabian lineages, resolving past admixture between and among populations, and uncovering the lineages that have been lost.

The MSY pattern in Arabian horses showed definite Arabian-specific sHGs, which point to linebreeding with selection on males beyond the documented period. On the other hand, the maternal lineages, explained by the mtDNA phylogenetic pattern, have indicated a high maternal diversity of the Arabian breed [28,50,57]. Therefore, the current Arabian horse breed is another example of differing variation of both maternal and paternal history in horses [58]. From a historical and traditional breeding point of view, the matrilineal side was and is still considered the major criterion to determine the purity of the Arabian horses. This work clearly shows that the paternal side is equally important, but a combined systematic analysis of both Y HTs and mtDNA is needed to determine the contrasting patterns of genetic variation in the two genetic compartments in the Arabian horse populations. Given the long history of maternal-oriented breeding practices in the places of breed origin and the small effective population size in the rest of the world, a more detailed comparison of mtDNA and Y HTs would be quite instructional.

## 4. Conclusions

In this study, we corroborated MSY haplotyping as a meaningful method for addressing population genetic, genealogical, and forensic questions in the recently established modern horse lineages. We present the next milestone for tracing stallion-mediated improvement and fine-scaled sire-line characterization. The determination of the Arabian and Thoroughbred MSY HT signatures now allows the tracing of their recent impact in any breed. However, further investigation is needed, and this should include enlarging the ascertainment panel, determining HT frequency distributions on a broader scale, and implementing faster-evolving markers in the analysis. In the future, MSY lineage determination can substantiate horse breeding in a way that contributes to the understanding of the historic development of breeds, supports decision-making in breed conservation, and can be used to validate the paternal ancestry in pedigree records.

## Figures and Tables

**Table 1:** Y chromosome haplotypes in occidental Arabian horse lines. Full information about the 145 males sampled (81 registered Arabians and 67 from other breeds), including the tail-male line, is given in Table S5. See Figure 2 for genetic relationships among the haplotypes.

Foundation Sire ^1^	Imported	Line Represented in Dataset via	Registered Arabians Sampled From ^2^	Breed Sampled ^3^	Y-Chromosomal Haplotype	Remarks
**Koheilan Adjuze db** Sbaa Anazeh	1885 Hungary	Piolun, 1934, Poland Jaszmak, 1928, Poland	Austria(1), Russia(5)	Trak(2) ShA(3)	Ao-aA1a* Ao-aA1a*	
**Mahmoud Mirza db** *Mukhalladiyah* Asad (Iraq)	India, Great Britain, Hungary	Jussuf I, 1962, Shagya Arabian		ShA(2)	Ao-aA1a*	
**Old Jellabi Speckled** (Bahrain)	Bahrain Foundation horse	Dhahmaan Alawwal, 1938, Bahrain	Austria(2), Poland(1)		Ao-aA1a*	
**Zarif** *Muniqi* Shammar Bedouins	1840 Germany	Rex II 372, 1941, Fredriksborg Horse Hermolin, 1937, Knabstrupper		FH(4) Ks(2)	Ao-aA1a* Ao-aA1a*	
**Zobeyni db** *Seglawi Jedran Ibn Sbeini* F’daan	Egyptian Foundation horse	Mahruss II, 1893, Egypt	Germany(1)		Ao-aA1a*	
**Ilderim db** *Saqlawi Jidran*	1900 Poland	Aquinor, 1951, Poland Maharadscha, 1957, Trakehner Doktryner, 1950, Poland	Poland(3) USA(1)	Trak(1), Wb(2) Le(1)	Ao-aA1a* Ao-aA1a4 Ta-b	subline-HT incongruence ^4^
**Saklawi I** *Saklawi Jidran Ibn Sudan*	Egyptian Foundation horse	Ansata Ibn Halima, 1958, Egypt Aswan, 1958, Egypt Galal, 1959, Egypt Habdan Enzahi, 1952, Egypt Morafic, 1952, Egypt	Egypt(1), Qatar(3), Poland(2), Syria(1) Iran(1), Poland(1), Qatar(1), Russia(1) Egypt(1), Germany(1) Austria(1), Egypt(3), Iran(2), Poland(5), Qatar(4)	ShA(1) ShA(1) ShA(2)	Ao-aA1a1 Ao-aA1a1 Ao-aA1a1 Ao-aA1a1 Ao-aA1a1	
**El Deree db** *Saqlawi Shaifi* Baqqara (Syria/Iraq)	Egyptian Foundation horse	Akhtal, 1968, Egypt	Qatar(2)		Ao-aA1a1	
**Siglavy db** ‚Schwarzenberg‘ *Saklawi*	1814 Austria	Siglavy Monterosa, 1907, Lipizzaner Siglavy Capriola III, 1940, Lipizzaner 21 Siglavy II, 1909, Shagya Arabian		Lip(7), Kl(1) Lip(4) ShA(1), Trak(2)	Ao-aA1a2 Ao-aA1a2a Ao-aA1b	subline-HT private HT
**Ibrahim db** *Saklawi Faliti* Banu Sakhr	1907 Poland	Negatiw, 1945, Russia Ferseyn, 1937, USA	Austria(1), Iran(2), Poland(2), Russia(2) United Arab Emirates(1)	PA(1), Pi(1)	Ao-aA1a3 Ao-aA1a3	
**Siglavy Bagdady db** *Saklawi* Ruala	1902 Hungary	Siglavy Bagdady VI, 1949, Babolna	Germany(1)	ShA(1), PA(1)	Ao-aA1a5	
**Dahoman db** *Dahman* Djelas Anazeh	1852 Hungary	Dahoman XVI, 1904, Shagya Arabian		ShA(1)	Ao-aA3	
**Jamil El Kebir db** *Saklawi Jidran* F‘daan	Egyptian Foundation horse	Anter, 1946, Egypt	Germany(1)		Ao-aD2	
**Krzyzyk db**	1876 Poland	Enwer Bey, 1923, Poland	Poland(2)		Ao-aD2	
**Mirage db** *Seglavi Jedran Dalia* Sbaa (Iraq)	1923 Great Britain	Bey Shah, 1976, USA	Qatar(1), Poland(2)		Ao-aD2	
**Kuhailan Haifi db** *Koheilan Haifi* Ruala	1931 Poland	Bask, 1956, Poland Celebes, 1949, Poland Wielki Szlem, 1938, POL	Poland(2), Qatar(1) Austria(1), Iran(1), Poland(1), United Arab Emirates(1)	PA(1) WB(1)	Ao-aD2 Ao-aD2 Ao-aD2	
**Hadban db** *Hadban Inzihi* S’baa	1897 Hungary	Habdan XI, 1954, Shagya Arabian		ShA(1)	Ao-aD2	
**Gazlan db** *Kuhaylan Tamri* Would Ali Anazeh (Iraq)	1852 Lipizza	Gazal VII, 1944, Shagya Arabian		ShA(6)	Ta*	
**O’Bajan db** *Ma’anaqi Sbaili* S’baa Anazeh	1885 Hungary	O’Bajan VII-4 530, 1936, Shagya Arabian O’Bajan X, 1929, Shagya Arabian		ShA(2) ShA(2)	Ta* Ta*	
**Shagya db** Beni Saher	1836 Hungary	Shagya IV, 1875, Shagya Arabian Shagya VII, 1877, Shagya Arabian Shagya XI, 1886, Shagya Arabian		ShA(3) ShA(2) ShA(3)	Ta-s Ta-s Ta-s	
**Mersuch db** *Hamdani Semri* Hazaim Pasha (Iraq)	1902 Hungary	Mersuch IV, 1936, Shagya Arabian		ShA(1)	Ta-b	
**Bairactar db** *Saklawi Jidran* (Syria)	1817 Germany	Arax, 1952, Poland Gwarny,1952	Poland(1), Qatar(1), Russia(1) Germany(2), Poland(1)		Ta-b Ta-bA	subline-HT
**Dahman Amir db** *Dahman Amir* Sultan Abdulhamid II (Turkey)	1902 Poland	Saludo, 1954, Spain	Qatar(1)	PA(1)	Ta-b	
**Seanderich db** *Saklawi Jidran* Shammar	1908 Spain	Tabal, 1952, Spain	Germany(1)		T2*	
**Kuhailan Afas db** Koheilan Afas (Bahrain)	1931 Poland	Comet, 1953, Poland	Germany(1), Poland(1)		Tb-oB1*	
**Latif db** *Hamdani Semri* Anazeh Fedan	1909 France	Baroud II, 1927, France Kann, 1927, France	Qatar(3), Russia(1) Russia(1)		Tb-oB1* Tb-oB1*	

^1^ Name of Foundation Sire, *Strain*, Bedouin tribe, or breeder (country). ^2^ The number of horses sampled is given in parenthesis. ^3^ The number of horses sampled is given in parenthesis. Abbreviations: Trakehner (Trak), Shagya Arabian (ShA), Fredriksborg Horse (FH), Knabstrupper (Ks), Warmblood (Wb), Lewitzer (Le), Lipizzaner (Lip), Kladruber (Kl),Partbred Arabian (PA), Pintabian (Pi). ^4^ Incongruence between tail-male line documentation and HT.

## Data Availability

NGS reads mapped to the LipY764 assembly of all samples analyzed in this paper are available at SRA archive PRJNA430351 (WGS data) and PRJNA787432 (TES data).

## Supplementary Materials

The following are available online at https://www.mdpi.com/article/10.3390/genes13020229/s1, Table S1: NGS Data information and MSY HTs; Table S2: Target enrichment bait regions on LipY764; Table S3: Information for haplotyping; Table S4: MSY Haplotypes on the genotyping scale; Table S5: Database with information on samples used for haplotyping; and Table S6: NGS results table. Figure S1: Backbone tree with HT-determining variants on branches.

## Appendix A

**Table A1:** Glossary.

Allele	Variant or Alternative form of the DNA Sequence at a Given Locus
clade	a branch on a phylogenetic tree that is formed from individuals of common descent; synonymous to ‘monophyletic group’
crown Haplogroup	very recently expanded horse MSY HG, predominant in modern breeds
founder/foundation sire	in this article this term is used to mean the earliest recorded paternal ancestor of an established sire line
genetic marker	a genetic marker is a DNA sequence with a known physical location on a chromosome that is polymorphic and thus informative for differentiating individuals
genotype	alleles possessed by an individual at one locus
haplotype (HT)	a set of DNA polymorphisms that are inherited together due to linkage; a Y-chromosomal haplotype is characterized by the allelic state at several markers
haplotyping	the laboratory process of determining the haplotype of an individual via the genotyping of appropriate markers
haplogroup (HG)	a monophyletic group of MSY HTs
indel	an insertion or deletion of bases in the genome that occurs at a specific genomic position
key variant	in this article, this term entitles the marker selected for haplotyping among markers with tautological readout
locus	a location in the genome; references to any sequence or genomic region, including non-coding regions
mitochondrial DNA (mtDNA)	circular, double-stranded DNA located in the matrix of a mitochondrion; the mtDNA is inherited uniparentally from mother to offspring
modern breed	in this article, this term is used for breeds that were developed or created within the westernized industry of horse breeding, spanning the recent 200 years; here it applies to Arabians, Thoroughbreds, Warmbloods, Central European and British Coldbloods, Central European and British Ponies, Baroque Type, Iberian, and New World breeds
most recent common ancestor (MRCA)	the sequence status from which all HT variations in a clade descend
male-specific region of the Y chromosome (MSY)	the non-recombining region of the Y chromosome which is inherited as a single linkage group from father to sons
patriline	the line of descent traced through the paternal side of the pedigree; synonymous with ‘male-tail line’
parsimony	a principle when drawing phylogenetic trees where the most likely tree is the one with the fewest evolutionary changes
pedigree	the record of descent of a horse
phylogeny	a branching tree showing the evolutionary relationships among a set of DNA sequences
sequence assembly	recreation of the original genome from the sequenced reads
short tandem repeats (STRs)	a tract of repetitive DNA in which short DNA motifs (ranging in length from one to six or more base pairs) are repeated; synonymous to ‘microsatellites’
single copy Y (scY) regions	well-explored regions of the MSY that are screenable with short-read data
single nucleotide variant (SNVs)	a variant of a single nucleotide that occurs at a specific genomic position
sire line	members of a sire line descend from the same foundation sire in their patriline (male-tail line); hence the breeding influence of a foundation sire is apparent from the sire line distribution range
subline HT	those HTs which emerged recently, within the pedigree supported timeframe, from a new mutation and are therefore unique markers for a particular stallion
target enriched sequencing	NGS sequencing of a specific (desired) regions of the genome
variant calling	identification of variation, mostly SNVs and Indels, present in sequence data by comparing NGS data from individuals with a reference sequence
Y chromosome	one of the two sex chromosomes; determines male sex in horses

## Appendix B

Explanatory information for creating a refined Y-chromosomal haplotype phylogeny.

**Table A2:** Versions of the NGS tools used in data analysis.

Tool	Reference	Version
AdapterRemoval	[30]	2.3.1
ReadTools	[31]	0.2.1.r_716422a3
bwa	[32]	0.7.17
samtools	[33]	1.10
GenomeAnalysisTK	[34]	3.7
bedtools	[35]	2.27.1
IGV	[36]	2.5.3
freebayes	[37]	1.3.2-46-g2c1e395
Network	[39]	10.2

## Appendix C

**Table A3:** Explanation of HG/HT nomenclature. HG/HT-determining variants are provided in Figure 2 and Table S3.

Major Crown Clades (*n* = 3—A, H, and T)	Haplogroups	Subhaplogroups and Haplotypes Detectable with Genotyping	Comments Regarding Nomenclature
Clade A was first described in an Arabian horse ‘A’			
	Ad-b		A draft: british
	Ad-h		A draft: heavy
	Am		A marchador
	Ao-aA		A original: arabian Autochthonous
		Ao-aA1a*, Ao-aA1a1, Ao-aA1a2, Ao-aA1a2a, Ao-aA1a3, Ao-aA1a4, Ao-aA1a5, Ao-aA1b, Ao-aA2, Ao-aA3	
	Ao-aD		A original: arabian Duelmener
		Ao-aD1, Ao-aD2	
			
	Ao-aM		A original: arabian Marwari
	Ao-n		A original: noriker
Clade H was first described in a Spanish (H) Sorraia horse			
	Hc		H china
	Hs		H spanish
		Hs-a	H spanish: autochthonous
		Hs-b	H spanish: barb
Clade T was first described in Thoroughbreds (T)			
	Ta		T arabian
		Ta*	
		Ta-s	T arabian: shagya
		Ta-b	T arabian: bairactar
		Ta-bA	
	Tb-d		T(horough)bred: darley
		Tb-dM	T(horough)bred: darley Mambrino
		Tb-dW	T(horough)bred: darley Whalebone
		Tb-dW1, Tb-dW2, Tb-dW3, Tb-dW4	
	Tb-o		T(horough)bred: other
		Tb-oB	T(horough)bred: other Byerley/Godolphin
		Tb-oB1*, Tb-oB1a, Tb-oB1b, Tb-oB1c, Tb-oB2, Tb-oB3*, Tb-oB3a*, Tb-oB3a1, Tb-oB3b*, Tb-oB3b1*, Tb-oB3b1a, Tb-oB3b1b, Tb-oB3b1c, Tb-oB4	
		Tb-oL	T(horough)bred: other Lipizzan
	Tk		T kladruber
	Tu		T ubiquitous
Non-crown Haplogroups			
I			Icelandic horse
N			Northern Europe
P			Przewalski
