# Supplementary figures and images for: Y-Chromosomal Insights into Breeding History and Sire Line Genealogies of Arabian Horses
Source: Genes (Basel). 2022 Jan 26;13(2):229. doi: 10.3390/genes13020229 (PMC8871751; doi:10.3390/genes13020229)

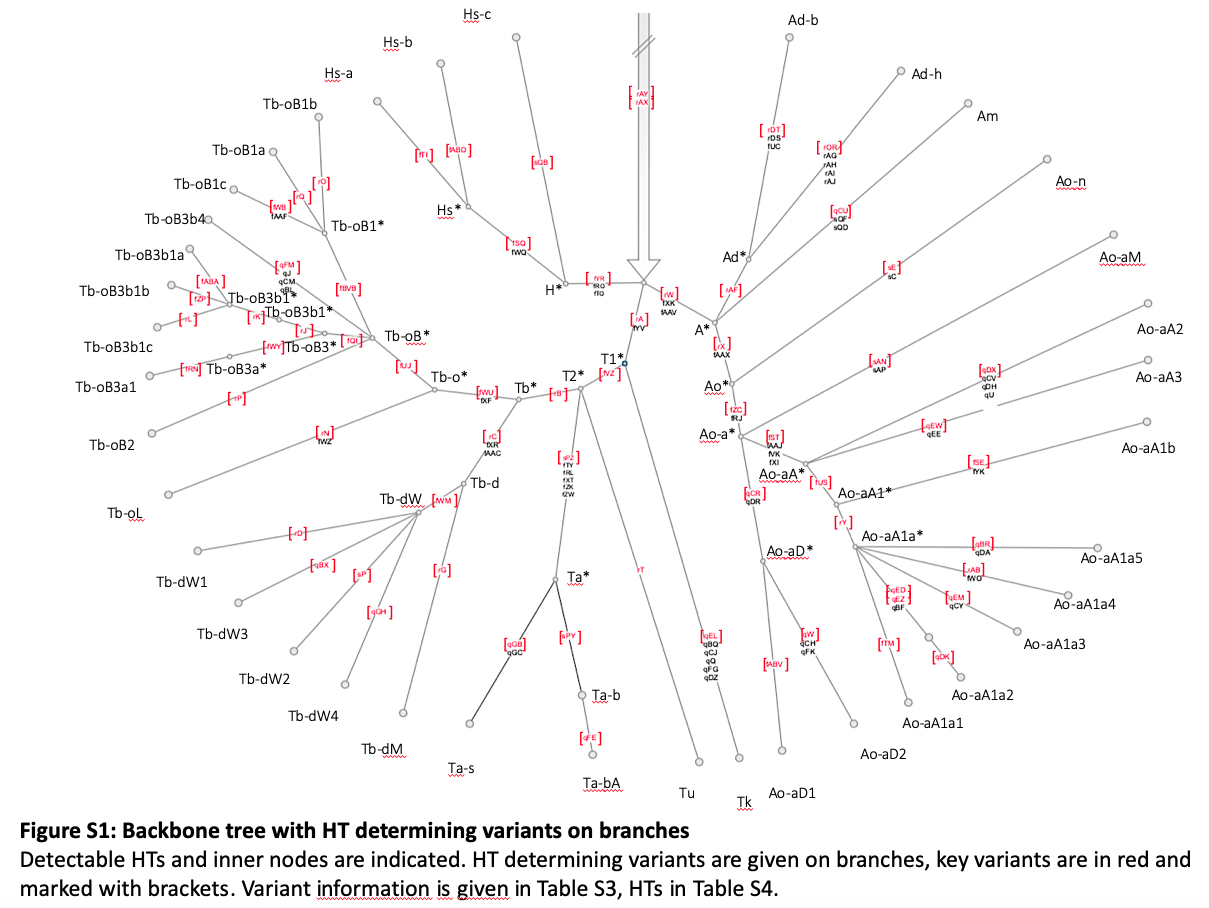

Supplement: Supplementary file 1 [file genes-13-00229-s001.zip › Figure S1_Backbone_tree2021220.jpg]
